# Supplementary material for: Machine learning models for segmentation and classification of cyanobacterial cells
Source: Photosynth Res. 2025 Feb 8;163(1):16. doi: 10.1007/s11120-025-01140-x (PMC11807057; doi:10.1007/s11120-025-01140-x)
Supplement: Supplementary file 1 — Supplementary file1 (DOCX 3376 KB) [file 11120_2025_1140_MOESM1_ESM.docx]

**Supplementary Information**

**S1: Additional equations for pixel-based performance**

1. **Precision**The precision score describes the proportion of correctly labeled “positives” (in our case, a true pixel in the predicted mask)

$$\text{Precision= }\frac{\text{True Positive}}{\text{True }\text{Positive+False}\text{ Positive}}$$

1. **Recall**The recall score describes how likely a positive classification (in our case, a true pixel in the predicted mask) is likely to be correct.

$$\text{Recall= }\frac{\text{True Positive}}{\text{True }\text{Positive+False}\text{ Negative}}$$

**Supplemental Table 1**. Description of the models used in this study, along with descriptions of the training datasets.

| Model name | Description | Training dataset | Reference |
| --- | --- | --- | --- |
| cyto2 | Cell cytoplasm segmentation model from Cellpose version 2.0. | Trained in two-channel images, where first channel is channel to segment and second is an optional nuclear channel | ^16^ |
| cyto3 | Cell cytoplasm segmentation model from Cellpose version 3.0. | 9 datasets; Trained to generate images which segment well from noisy images | ^18^ |
| bact-phase-omni | Bacterial segmentation model from Omnipose | Phase contrast images of assorted bacterial species with diverse morphologies and optical characteristics, 27,500 total cells | ^19^ |
| cypose-7002 | Fine-tuned model for segmentation of PCC 7002 cells | 6 movies of PCC 7002, 413 frames, 35,000 total cells | This study |
| cypose-7002-scratch | Scratch-trained model for segmentation of PCC 7002 cells | 18 movies of PCC 7002 WT, *∆murA, ∆ftsh1-4, ∆pdbH, ∆ftsZ,* 2271 frames, 125,040 total cells. | This study |
| cypose-33047 | Fine-tuned model for segmentation of filamentous Anabaena cells | 4 movies of ATCC 33047, 233 frames, 68411 total cells | This study |
| cyclass-7002 | Cell classification network | 9 movies of PCC 7002, 736 frames, 55,695 total cells | ^26^ |


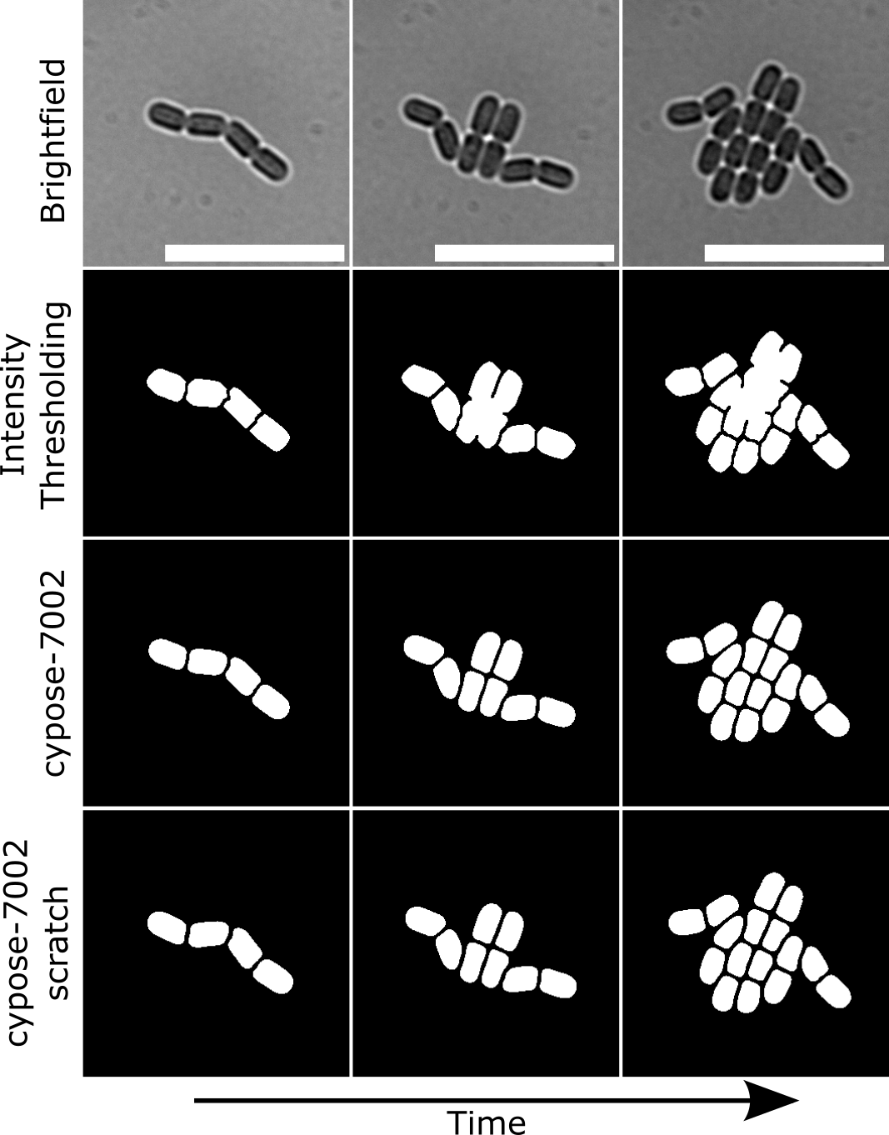


**Supplemental Figure 1:** Comparison of segmentation of 7002 between intensity-based thresholding, cypose-7002 and cypose-7002-scratch. Scale bars indicate 15 µm.


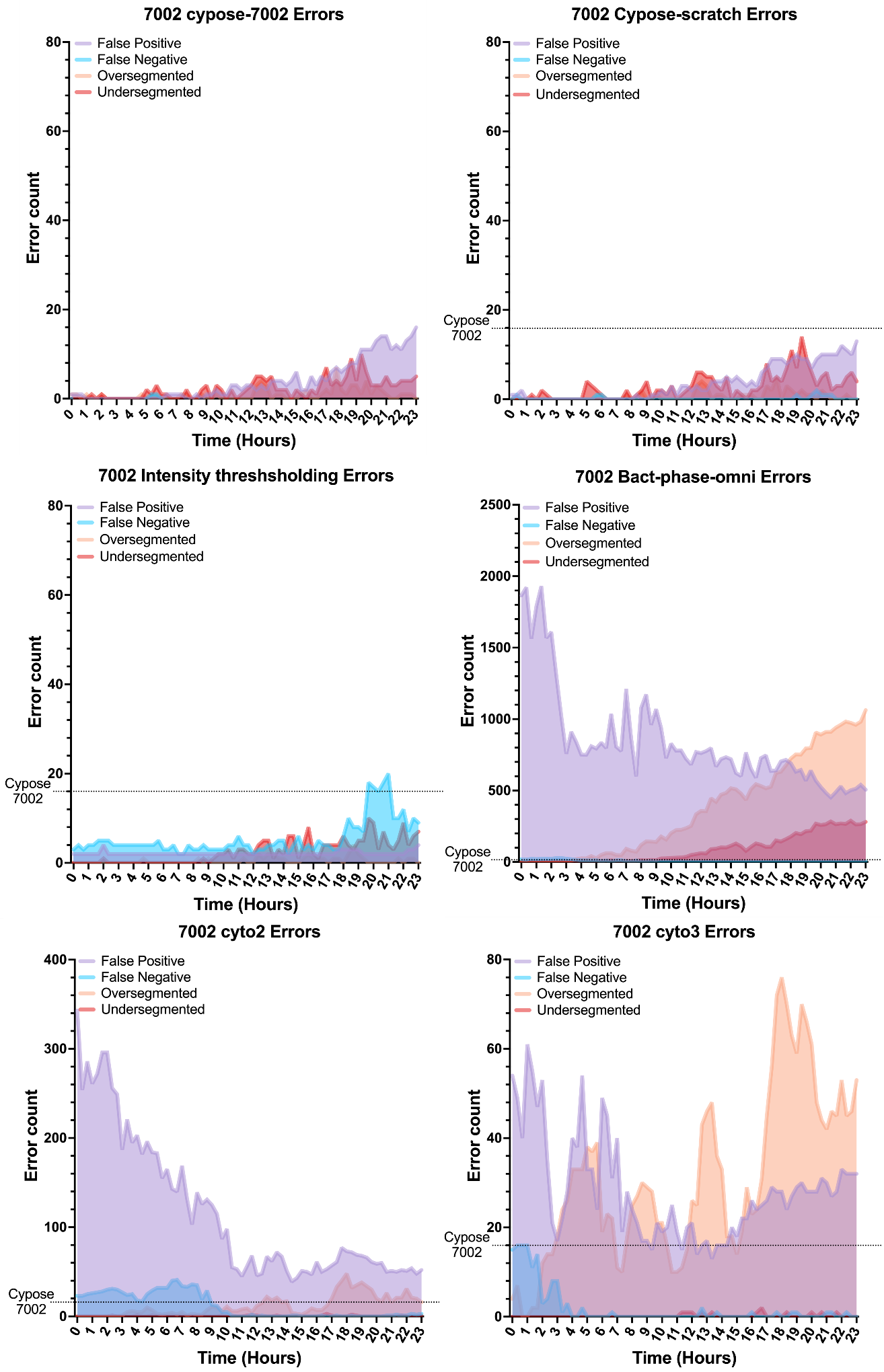


**Supplemental Figure 2:** Segmentation error over time of the PCC 7002 benchmark. The max number of errors for cypose-7002 is marked on the other graphs to provide easier relative comparison.


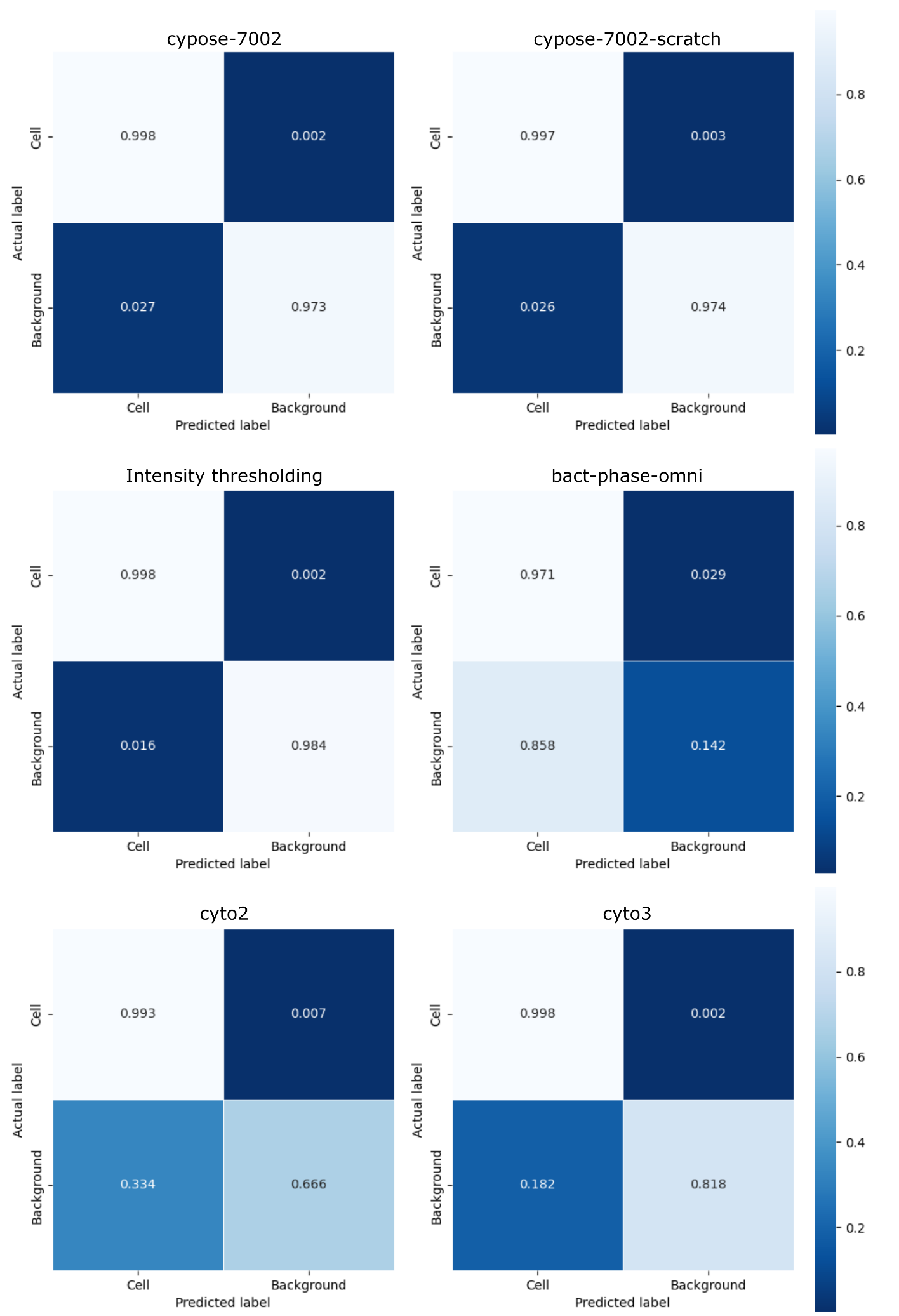


**Supplemental Figure 3:** Confusion matrixes for segmentation models on the PCC 7002 benchmark.


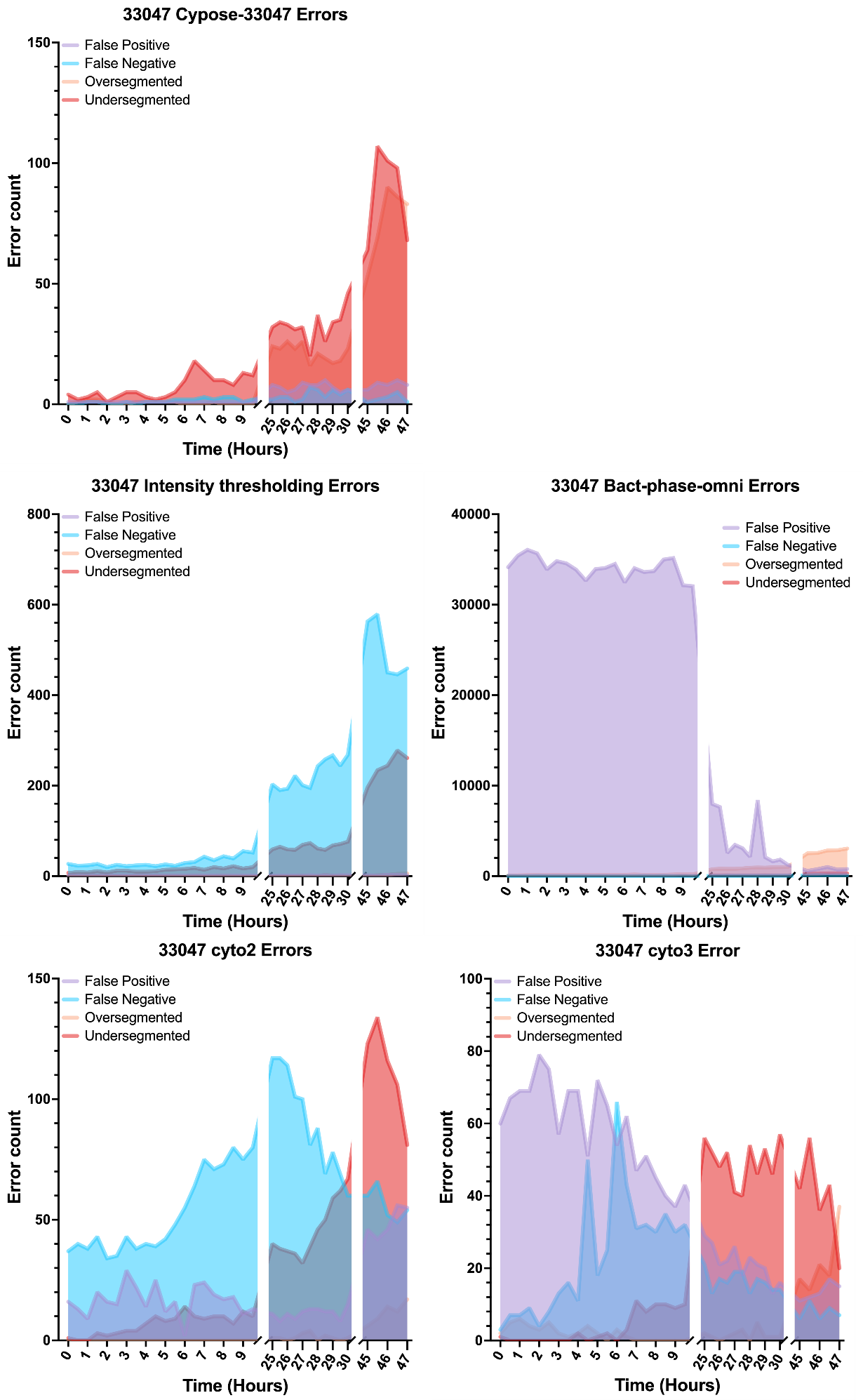


**Supplemental Figure 4:** Segmentation errors over time of the ATCC 33047 benchmark.


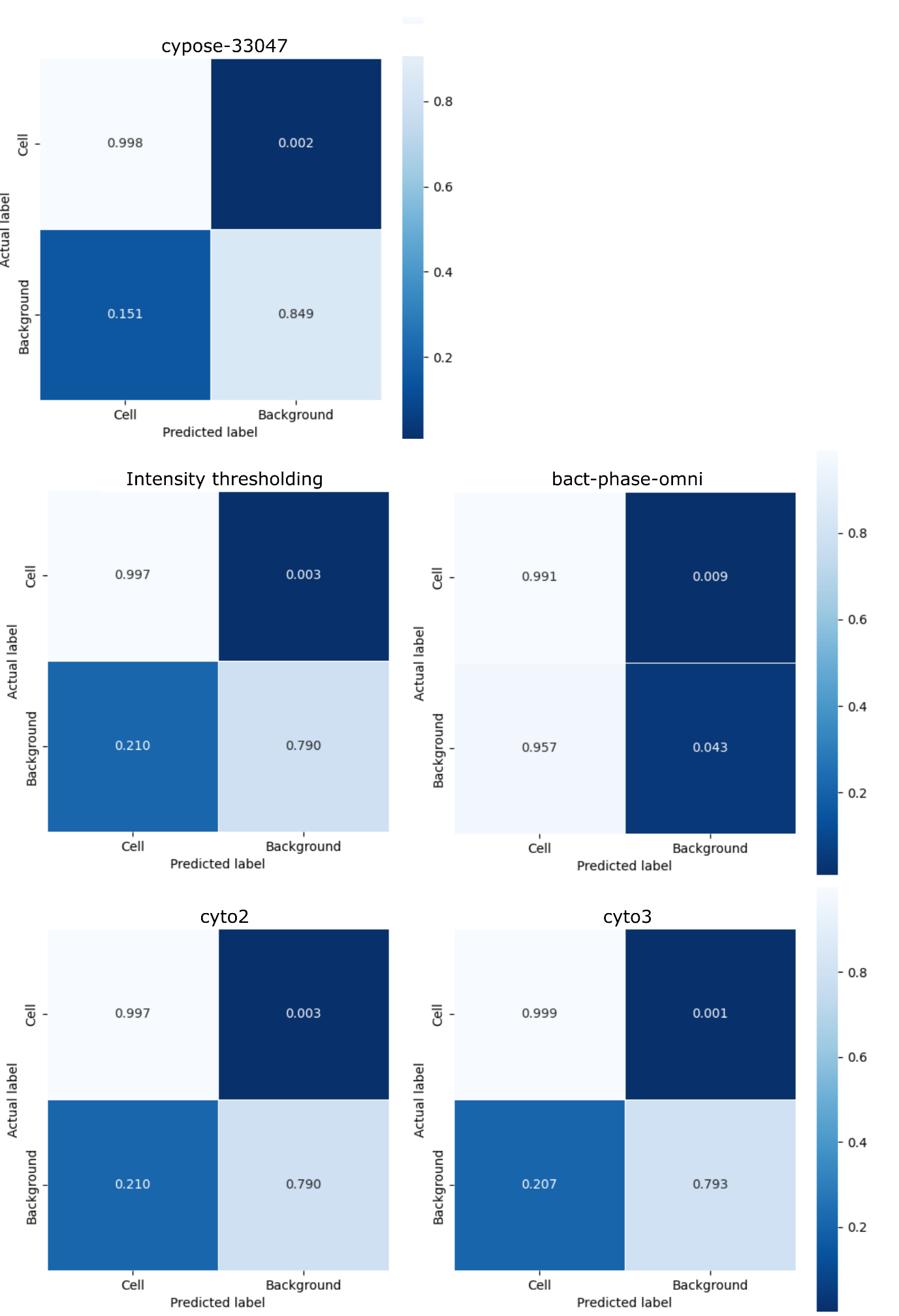


**Supplemental Figure 5:** Confusion matrixes for segmentation models on the ATCC 33047 benchmark.


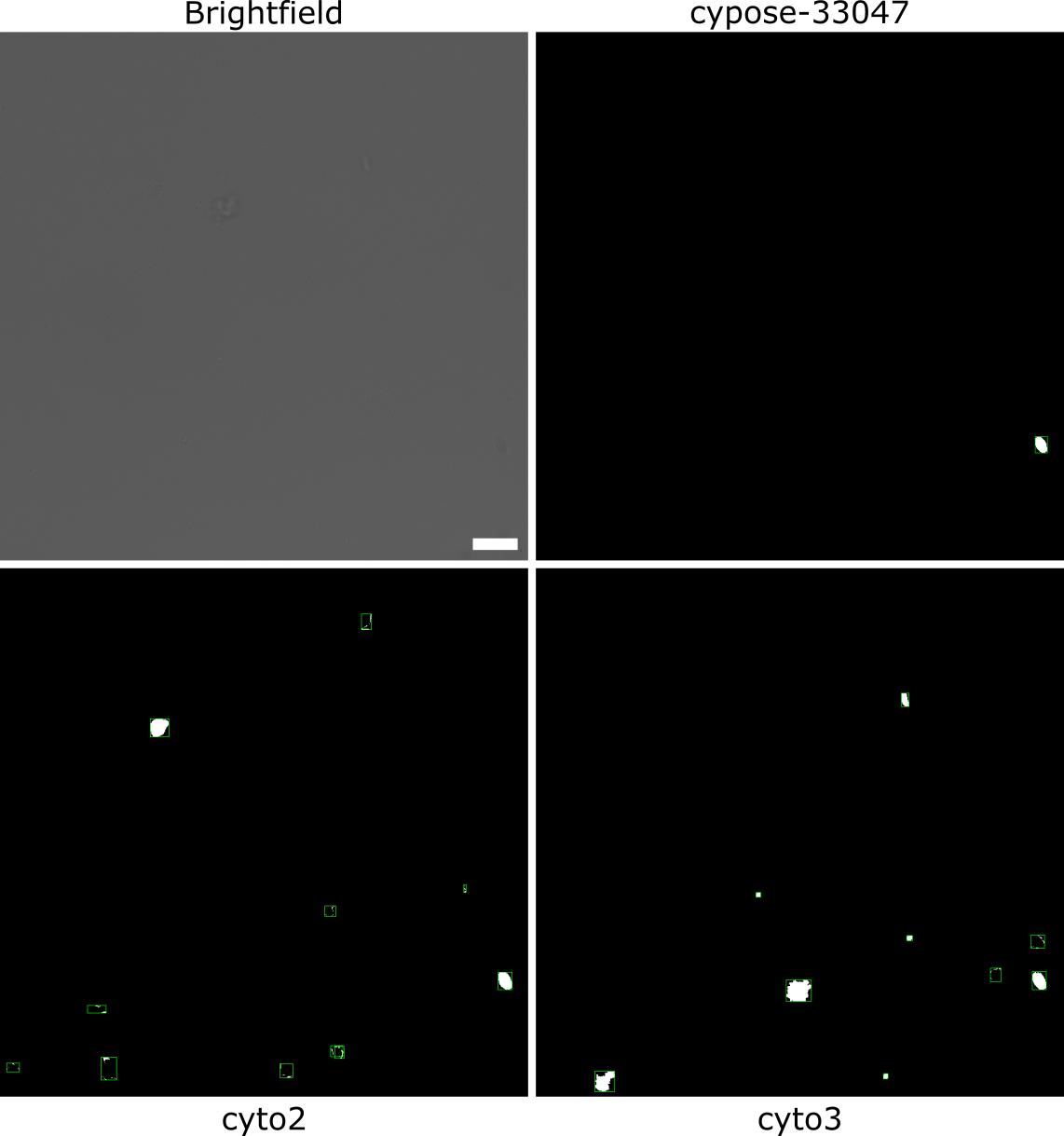


**Supplemental Figure 6:** ATCC 33047 segmentation comparisons showing false positive segmentation of debris and background in an area without cells present. False positives (additional objects) errors in each mask are highlighted by a green box.


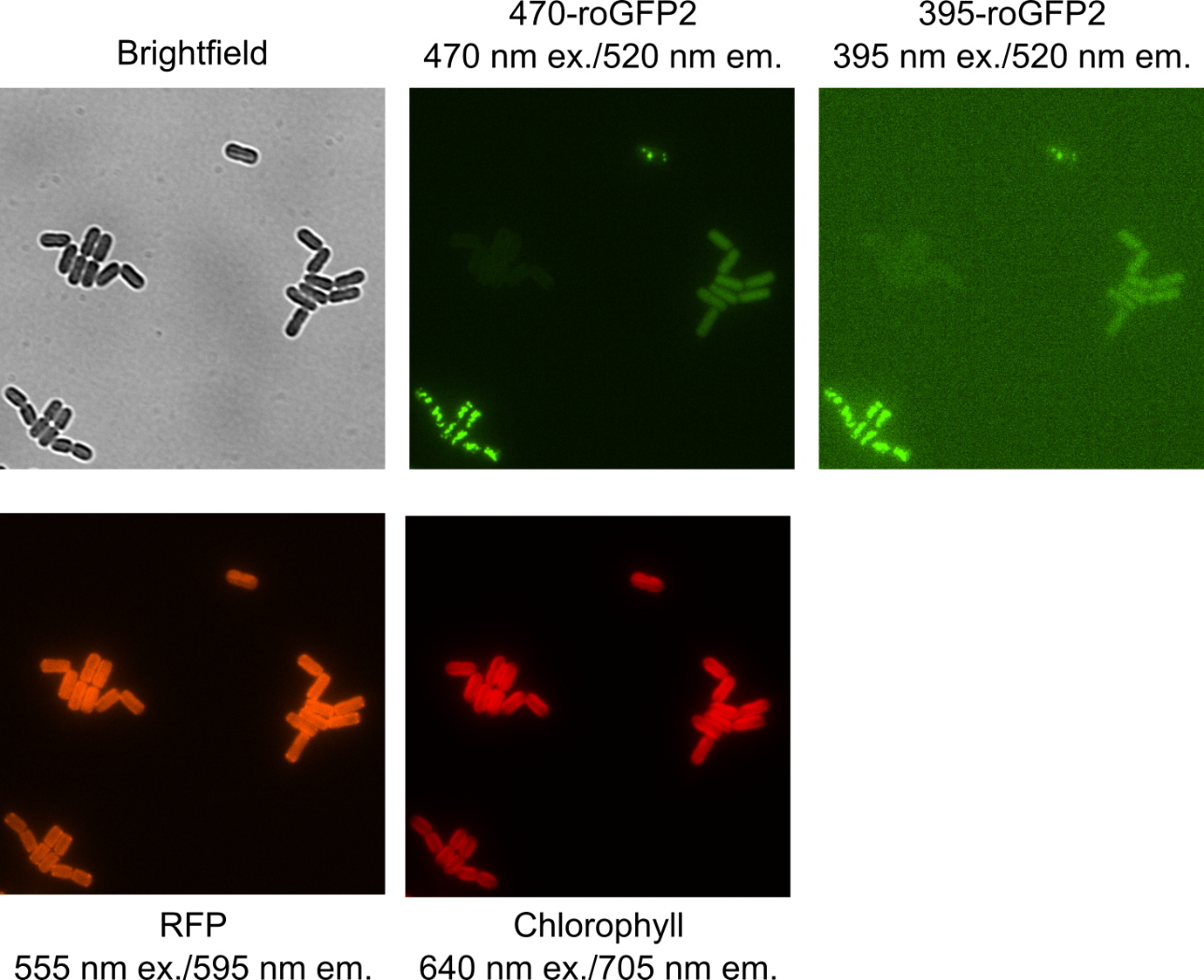


**Supplemental Figure 7:** All channels of the input image used to train the classifier. The channels were combined into a single matrix and used as input to the classifier. Note that the roGFP2 fluorophore is a ratiometric redox sensor^26^.


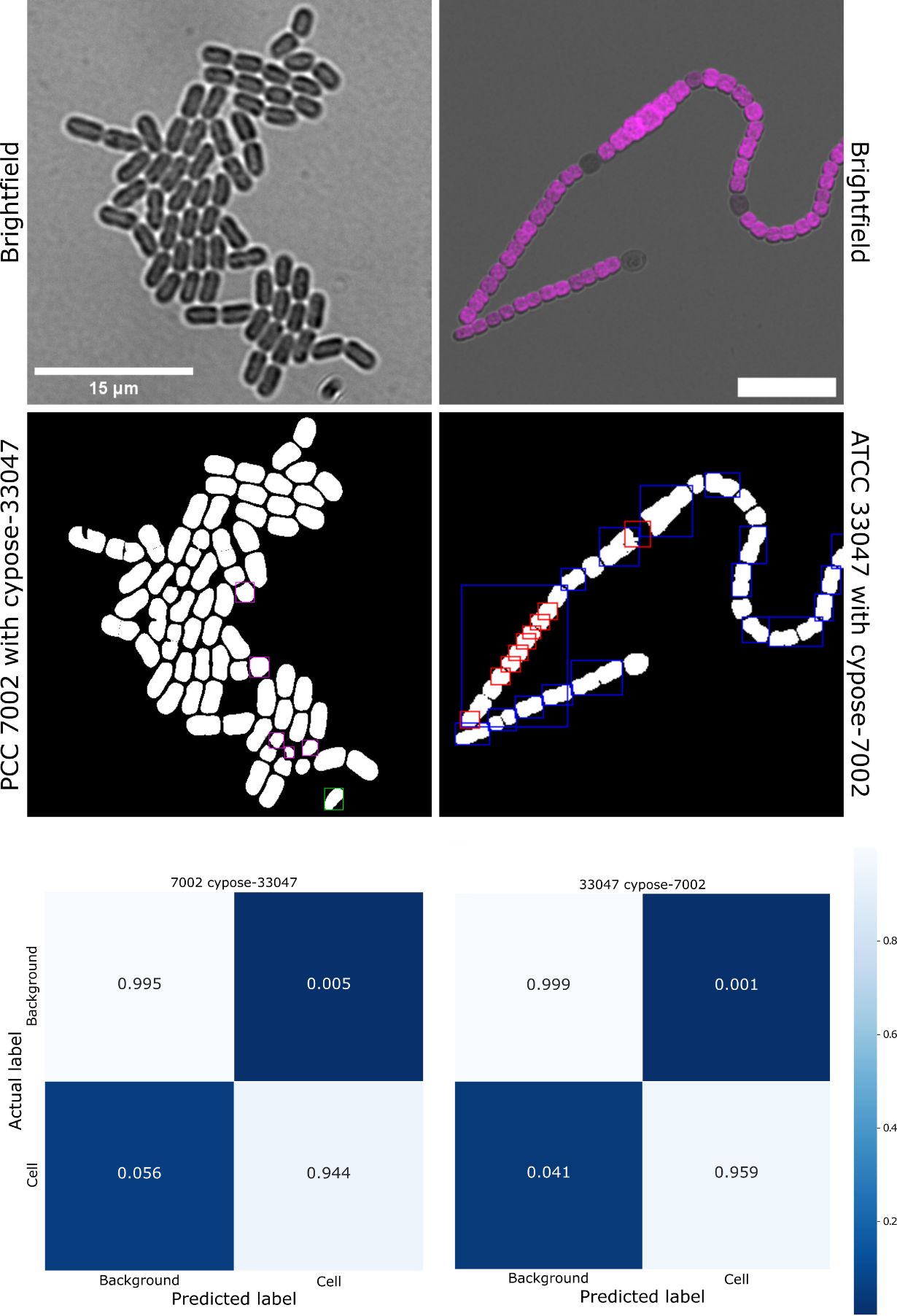


**Supplemental Figure 8:** Segmentation results and confusion matrices of cross application of the fine-tuned models to the opposite species (cypose-33047 segmenting PCC 7002 and cypose-7002 segmenting ATCC 33047). Errors in each mask are highlighted by a box: undersegmentation in blue, oversegmentation in magenta, false positives (additional objects) in green, and false negatives (missing objects) in red.
